# Retraction: The PI3K/Akt and NF-κB signaling pathways are involved in the protective effects of *Lithocarpus polystachyus* (sweet tea) on APAP-induced oxidative stress injury in mice

**DOI:** 10.1039/d2ra90100e

**Published:** 2022-10-07

**Authors:** Jia-yu Yang, Yu-te Zhong, Wei-nan Hao, Xiang-xiang Liu, Qiong Shen, Yan-fei Li, Shen Ren, Zi Wang, Wei Li, Li-Chun Zhao

**Affiliations:** College of Chinese Medicinal Materials, Jilin Agricultural University Changchun 130118 China liwei7727@126.com +86-431-84533304 +86-431-84533304; College of Pharmacy, Guangxi University of Chinese Medicine Nanning 530200 China hyzlc@163.com

## Abstract

Retraction of ‘The PI3K/Akt and NF-κB signaling pathways are involved in the protective effects of *Lithocarpus polystachyus* (sweet tea) on APAP-induced oxidative stress injury in mice’ by Jia-yu Yang *et al.*, *RSC Adv.*, 2020, **10**, 18044–18053. https://doi.org/10.1039/D0RA00020E.

The Royal Society of Chemistry hereby wholly retracts this *RSC Advances* article due to concerns with the reliability of the data.

In Fig. 3B, part of the ‘APAP+STL-E (50 mg/kg)’ panel is duplicated in the ‘APAP+STL-E (100 mg/kg)’ panel.

In Fig. 4A, the first two bands of the ‘p-Akt’ panel, when rotated 180°, are identical to the first two bands of the ‘Cleaved-caspase 9’ panel.

The authors were asked to provide the raw data for this article, but did not respond. Given the significance of the concerns about the validity of the data, and the lack of raw data, the findings presented in this article are not reliable.

The authors were informed but have not responded to any correspondence regarding the retraction.

Signed: Laura Fisher, Executive Editor, *RSC Advances*.

Date: 18th August 2022.

## Supplementary Material

